# Hypoxia dynamics on FMISO-PET in combination with PD-1/PD-L1 expression has an impact on the clinical outcome of patients with Head-and-neck Squamous Cell Carcinoma undergoing Chemoradiation

**DOI:** 10.7150/thno.48392

**Published:** 2020-07-23

**Authors:** Alexander Rühle, Anca-L. Grosu, Nicole Wiedenmann, Michael Mix, Raluca Stoian, Gabriele Niedermann, Dimos Baltas, Martin Werner, Wolfgang A. Weber, Gian Kayser, Nils H. Nicolay

**Affiliations:** 1 Department of Radiation Oncology, Medical Center - University of Freiburg, Faculty of Medicine, University of Freiburg, Freiburg, Germany.; 2 German Cancer Consortium (DKTK), Partner Site Freiburg and German Cancer Research Center (DKFZ), Heidelberg, Germany.; 3 Department of Nuclear Medicine, Medical Center - University of Freiburg, Faculty of Medicine, University of Freiburg, Freiburg, Germany.; 4 Department of Nuclear Medicine, Technical University of Munich, Munich, Germany.; 5 Institute of Surgical Pathology, Department of Pathology, Medical Center - University of Freiburg, Faculty of Medicine, University of Freiburg, Freiburg, Germany.

**Keywords:** head-and-neck cancer, PD-L1, hypoxia, FMISO PET, radiotherapy

## Abstract

Tumor-associated hypoxia influences the radiation response of head-and-neck cancer (HNSCC) patients, and a lack of early hypoxia resolution during treatment considerably deteriorates outcomes. As the detrimental effects of hypoxia are partly related to the induction of an immunosuppressive microenvironment, we investigated the interaction between tumor hypoxia dynamics and the PD-1/PD-L1 axis in HNSCC patients undergoing chemoradiation and its relevance for patient outcomes in a prospective trial.

**Methods:** 49 patients treated with definitive chemoradiation for locally advanced HNSCC were enrolled in this trial and received longitudinal hypoxia PET imaging using fluorine-18 misonidazole ([^18^F]FMISO) at weeks 0, 2 and 5 during treatment. Pre-therapeutic tumor biopsies were immunohistochemically analyzed regarding the PD-1/PD-L1 expression both on immune cells and on tumor cells, and potential correlations between the PD-1/PD-L1 axis and tumor hypoxia dynamics during chemoradiation were assessed using Spearman's rank correlations. Hypoxia dynamics during treatment were quantified by subtracting the standardized uptake value (SUV) index at baseline from the SUV values at weeks 2 or 5, whereby SUV index was defined as ratio of maximum tumor [^18^F]FMISO SUV to mean SUV in the contralateral sternocleidomastoid muscle (*i.e.* tumor-to-muscle ratio). The impact of the PD-1/PD-L1 expression alone and in combination with persistent tumor hypoxia on locoregional control (LRC), progression-free survival (PFS) and overall survival (OS) was examined using log-rank tests and Cox proportional hazards models.

**Results:** Neither PD-L1 nor PD-1 expression levels on tumor-infiltrating immune cells influenced LRC (HR = 0.734; *p* = 0.480 for PD-L1, HR = 0.991; *p* = 0.989 for PD-1), PFS (HR = 0.813; *p* = 0.597 for PD-L1, HR = 0.796; *p* = 0.713 for PD-1) or OS (HR = 0.698; *p* = 0.405 for PD-L1, HR = 0.315; *p* = 0.265 for PD-1). However, patients with no hypoxia resolution between weeks 0 and 2 and PD-L1 expression on tumor cells, quantified by a tumor proportional score (TPS) of at least 1%, showed significantly worse LRC (HR = 3.374, *p* = 0.022) and a trend towards reduced PFS (HR = 2.752, *p* = 0.052). In the multivariate Cox regression analysis, the combination of absent tumor hypoxia resolution and high tumoral PD-L1 expression remained a significant prognosticator for impaired LRC (HR = 3.374, *p* = 0.022). On the other side, tumoral PD-L1 expression did not compromise the outcomes of patients whose tumor-associated hypoxia declined between week 0 and 2 during chemoradiation (LRC: HR = 1.186, *p* = 0.772, PFS: HR = 0.846, *p* = 0.766).

**Conclusion:** In this exploratory analysis, we showed for the first time that patients with both persistent tumor-associated hypoxia during treatment and PD-L1 expression on tumor cells exhibited a worse outcome, while the tumor cells' PD-L1 expression did not influence the outcomes of patients with early tumor hypoxia resolution. While the results have to be validated in an independent cohort, these findings form a foundation to investigate the combination of hypoxic modification and immune checkpoint inhibitors for the unfavorable subgroup, moving forward towards personalized radiation oncology treatment.

## Introduction

With around 650,000 new diagnoses and almost 400,000 deaths per year, head-and-neck squamous cell carcinoma (HNSCC) constitutes a prevalent malignancy with high morbidity and mortality, demonstrated by a 5-year survival rate between 40% and 70% depending on tumor stage and localization 1, 2. Locoregional treatment strategies commonly comprise either surgery or radiotherapy, and for locally advanced HNSCCs, radiation therapy in combination with chemotherapy constitutes a treatment standard.

Tumor-associated hypoxia is known to be a crucial factor regarding treatment outcomes of HNSCC patients undergoing chemoradiation, as hypoxia increases resistance to ionizing radiation 3-5. Various approaches to monitor tumor hypoxia during chemoradiation have been studied, including histological analyses, pO2 polarography and different imaging modalities 6-11. Fluorine-18 misonidazole positron emission tomography ([^18^F]FMISO PET) has proven to be a reliable imaging method for non-invasive monitoring of tumor hypoxia 12-15. It has been shown in previous studies that early hypoxia resolution during the course of chemoradiation has a major impact on treatment response compared to baseline hypoxia 6, 16, 17. The visualization of tumor hypoxia offers the possibility to integrate this important biological parameter for tumor radioresistance in the treatment planning, allowing for tumor control probability calculations and dose painting strategies for hypoxic subvolumes, which may help to move forward towards personalized radiation treatment in the future 18-20. However, escalation of radiation dose to hypoxic subvolumes, simultaneous administration of hypoxia modifiers, hyperbaric oxygen or hyperthermia treatment are not regularly used in the clinical routine.

Based on *in vitro* and animal experiments, there is cumulating evidence for an interaction between tumor-associated hypoxia and the immune system within the intratumoral microenvironment 21, 22. For instance, hypoxia has demonstrated to promote the release of immunosuppressive cytokines such as interleukin-10 and TGF-ß, to increase the expression of the programmed cell death ligand, PD-L1 both on immune cells and tumor cells, and to elevate the number of immunosuppressive immune cells including regulatory T cells, myeloid derived suppressor cells and tumor-associated macrophages 23-25. Programmed cell death protein‐1 (PD-1) and its ligand, PD‐L1, are known to be key factors by which cancer cells evade the anti-tumor activity of the immune system. Immune checkpoint inhibitors targeting PD-1 such as nivolumab and pembrolizumab have shown promising results in recurrent or metastatic HNSCC patients and have therefore gained approval for these indications 26-30. The upregulation of PD-L1 under hypoxic conditions gives a rationale to investigate the effectiveness of checkpoint inhibitors as treatment escalation strategy for patients with persistent tumor hypoxia during chemoradiation.

The current exploratory analysis is based on a prospective hypoxia imaging trial and intended to examine the interaction between the PD-1/PD-L1 axis and tumor hypoxia dynamics on FMISO-PET/CT during the course of chemoradiation in patients with locally advanced HNSCC.

## Methods

### Patient treatment

The study was registered in the German Clinical Trial Register (DRKS00003830) and was conducted in accordance with the Declaration of Helsinki (revised version of 2008). The Independent Ethics Committee of the University of Freiburg (reference no. 479/12) approved the trial in advance, and written informed consent was obtained from all patients prior to enrolment in this trial.

49 patients with locally advanced and histologically confirmed HNSCC were enrolled in this prospective imaging trial. Patients had a median age of 60 years (range 34 to 78 years) and were mostly male (n = 44; 89.8%). Detailed patient characteristics were reported previously and are summarized in **Table S1** 31. Patients underwent definitive chemoradiation with intensity-modulated radiotherapy and received a cumulative dose of 70 Gy in 35 fractions to the high-risk planning target volume (PTV) and 50 Gy in 25 fractions to the low-risk PTV. Cisplatin was administered in 3 cycles (100 mg/m² body surface area in weeks 1, 4 and 7) during radiotherapy.

### Imaging

**Figure 1:**
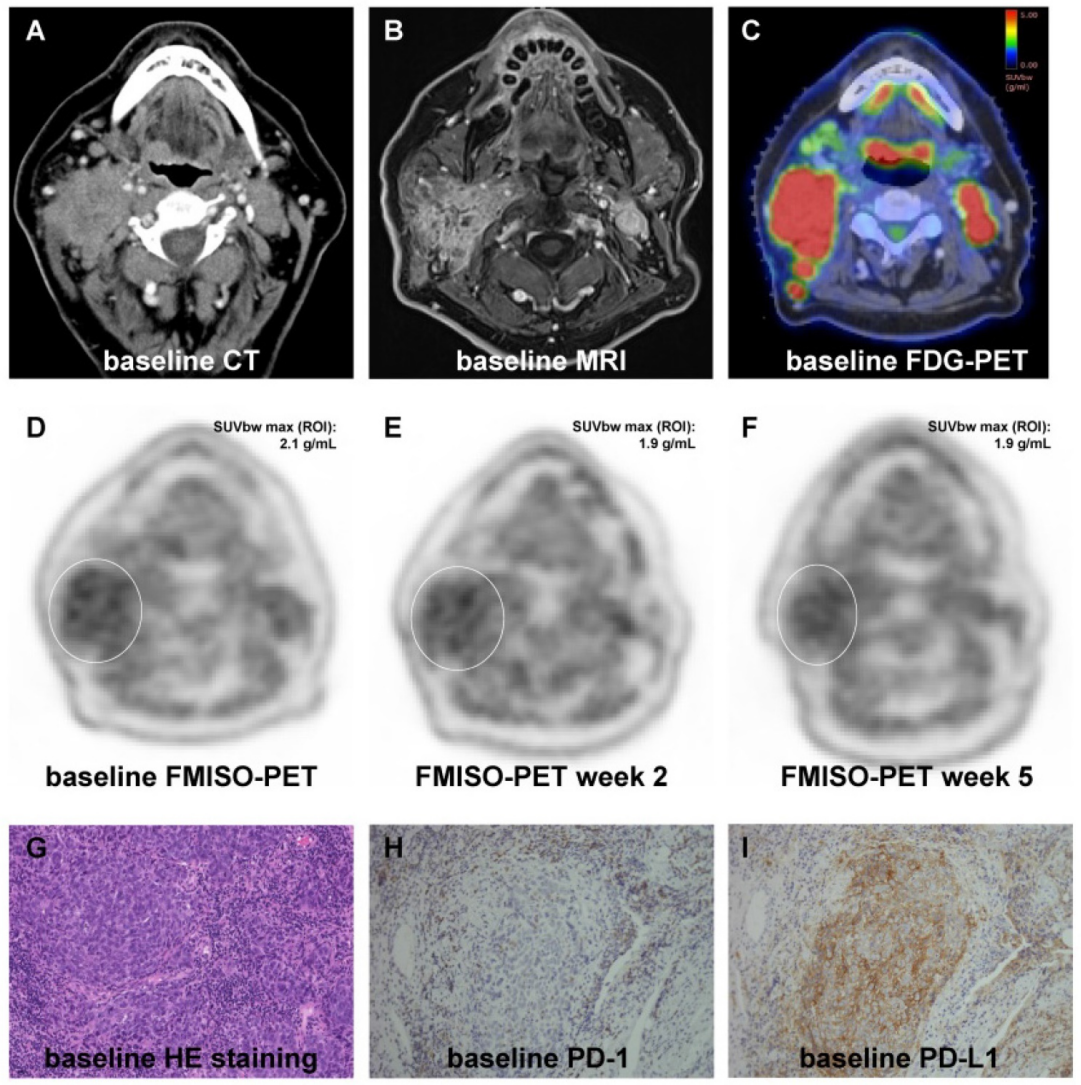
Representative images showing the dynamics of hypoxia resolution during chemoradiation as well as representative immunohistochemical stainings. (**A-C**) Pre-therapeutic CT (A), MRI (B) and [^18^F]FDG PET (C) images showing an oropharyngeal carcinoma with multiple confluent cervical lymph nodes, cT3 cN2c cM0. (**D-F**) [^18^F]FMISO PET at weeks 0 (D), 2 (E) and 5 (F) showing moderate pre-therapeutic hypoxia with a slight decrease during chemoradiation. (**G-I**) Representative HE staining (G) of the tumor sample as well as pre-therapeutic immunohistochemical staining for PD-1 (H) and PD-L1 (I) demonstrating high expression of PD-L1 and PD-1. 20x objective magnification.

All patients received computed tomography (CT) and magnetic resonance imaging (MRI) scans as part of the radiotherapy planning procedures as well as PET imaging with ^18^F-fluorodeoxyglucose ([^18^F]FDG) and [^18^F]FMISO tracers prior to treatment (**Figure 1**). Additionally, [^18^F]FMISO PET/CT scans were repeated in weeks 2 and 5 during treatment. PET imaging was performed as described earlier 31, 32. In short, 3.7 MBq/kg [^18^F]FMISO was injected to a maximum activity of 370 MBq, and hypoxia imaging was carried out in radiation treatment position using a thermoplastic head immobilization mask. A mutual information algorithm was used for co-registering PET images with the corresponding planning CTs 33.

Contouring of the gross tumor volume (GTVs) was conducted manually on the [^18^F]FDG-PET-MRI co-registered images, in which a PET threshold of 40% of the maximum SUV within the tumor was used 17. Subvolumes within the GTV were defined as hypoxic if the ratio of maximum tumor [^18^F]FMISO SUV to mean SUV in the contralateral sternocleidomastoid muscle (*i.e.* tumor-to-muscle ratio) was above 1.4. The used ratio has previously been validated in this study cohort 17, 34. Hypoxia dynamics during the course of chemoradiation treatment (ΔSUV index) were quantified by subtracting the SUV index at baseline from the SUV values at weeks 2 or 5.

### Immunohistochemistry

Tumor samples were fixed with formalin and embedded in paraffin according to institutional protocols. The embedded tumors were sectioned at 2 µm thickness and mounted on coated glass slides (Langenbrinck, Emmendingen, Germany) prior to deparaffinization and rehydration using descending graded ethanol concentrations. Heat-induced antigen retrieval was performed, and endogenous peroxidase activity was blocked with H_2_O_2_. Antigen complexes were visualized by an Envision Flex Kit combined with a mouse linker (DAKO) using horseradish peroxidase-diaminobenzidine (HRP-DAB) reaction, and cell nuclei were counterstained by hematoxylin. **Table S2** shows details of primary antibodies and antigen retrieval. P16 overexpression was used as a surrogate parameter for the HPV status, and p16 overexpression was defined for HNSCCs that exhibited more than 70% of cells with strong nuclear and/or cytoplasmatic staining.

The expression of the tissue hypoxia markers CAIX and HIF1α were examined semi-quantitatively using the H-score. The staining intensity (0 = no staining, 1 = weak, 2 = moderate, 3 = strong) was assessed for all viable tumor cells, and the H-score (range 0 - 300) was calculated as the sum of the tumor cell percentages of the different staining intensities multiplied by their specific intensity scores.

CD34 stainings were conducted to examine the microvessel density. For quantification, stainings were divided into 3 categories: 1 = only larger blood vessels in the stroma with no contact to HNSCCs; 2 = smaller vessels in the stroma with contact to tumor cells, 3 = small vessels intermingling with tumor cells at the border between tumor and stroma.

Both PD-1 and PD-L1 expression was divided into 3 groups based on the number of immune cells (lymphocytes, dendritic cells, macrophages, myeloid-derived suppressor cells) demonstrating PD-1 or PD-L1 expression per high power field (HPF): 0 = 0-20, 1 = 20-100, 2 = 100 and more positive immune cells (notably, there was no PD-1 expression = 2 in our cohort). PD-L1 expression on tumor cells was quantified using the tumor proportional score (TPS), which is the percentage of viable tumor cells showing partial or complete staining for PD-L1.

### Statistical analyses

Overall survival (OS) was calculated from the completion of treatment to death from any cause, progression-free survival (PFS) was quantified as the interval between treatment completion and disease progression or death of any cause. Locoregional control (LRC) was defined as the absence of any progression of the primary tumor or cervical lymph nodes. LRC, PFS and OS were calculated using the Kaplan-Meier method, and log-rank tests were used for testing differences between the Kaplan-Meier curves. Both univariate and multivariate Cox regression analyses were conducted in order to evaluate the influence of clinical parameters on the LRC and PFS of HNSCC patients. Parameters which proved to be significant in the univariate analysis were included in the multivariate Cox regression, in which a backward stepwise Cox regression with likelihood ratio tests was conducted. Spearman's rank correlation analyses were carried out to test associations between tissue hypoxia markers, PD-L1 status and tumor hypoxia dynamics. Potential differences between the PD-1 expression subgroups regarding their SUV index values were examined using unpaired t-tests. Prior to performing the t-tests, a Levene's test was conducted to test for homogeneity of variances, and a Shapiro-Wilk test was used to analyze normality of both groups. In order to reveal potential differences regarding the immune cells' PD-1 and PD-L1 expression as well as the tumoral PD-L1 expression (TPS) in dependence of the HPV status, chi-square tests (for PD-1, as only 2 category values exist) and Mann-Whitney-U tests (for PD-L1 and TPS) were used. *P*-values below 0.05 were considered statistically significant. All statistical analyses were carried out using IBM SPSS Statistics software version 25 (IBM, Armonk, NY, USA).

## Results

### Neither PD-L1 nor PD-1 expression on intratumoral immune cells influences the oncological outcomes after chemoradiation

**Figure 2:**
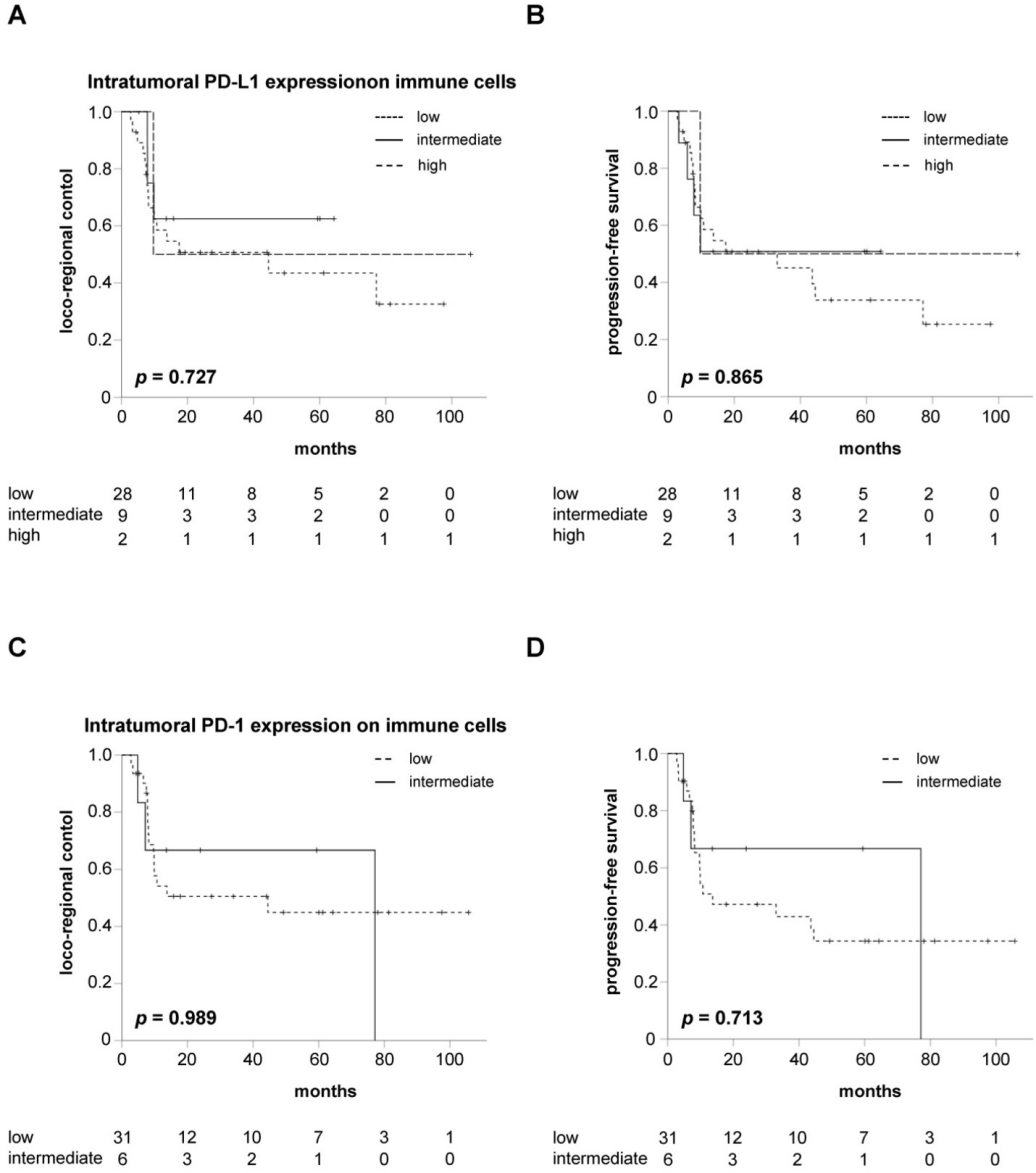
Neither PD-L1 nor PD-1 expression on intratumoral immune cells influences LRC or PFS in HNSCC patients undergoing chemoradiation. LRC (**A**) and PFS (**B**) in HNSCC patients undergoing chemoradiation in dependence of the intratumoral immune cells' PD-L1 expression in pre-therapeutic tumor biopsies. LRC (**C**) and PFS (**D**) in HNSCC patients stratified by the expression levels of intratumoral PD-1. P values are derived from log-rank tests.

Kaplan-Meier curves showed no significant differences between patients with varying expression levels of PD-L1 on intratumoral immune cells regarding LRC (*p* = 0.727), PFS (*p* = 0.865) or OS (*p* = 0.274) in our cohort (**Figure 2A-B, Figure S1A**). In line with the Kaplan-Meier analyses, Cox analyses revealed no difference regarding LRC (HR = 0.734, 95% CI 0.311 - 1.731, *p* = 0.480), PFS (HR = 0.813, 95% CI 0.377 - 1.752, *p* = 0.597) and OS (HR = 0.698, 95% CI 0.299 - 1.629, *p* = 0.405) for HNSCC patients with increased pre-therapeutic PD-L1 expression on intratumoral immune cells.

Similarly, LRC and PFS did not differ between patients with intermediate or low intratumoral PD-1 expression on immune cells (*p* = 0.989 for LRC, *p* = 0.713 for PFS) (**Figure 2C-D**). In line with this observation, OS was comparable between both groups (*p* = 0.239) (**Figure S1B**). Cox regression analyses demonstrated a HR for increased PD-1 expression on intratumoral immune cells of 0.991 (95% CI 0.287 - 3.428, *p* = 0.989), 0.796 (95% CI 0.234 - 2.704, *p* = 0.713) and 0.315 (95% CI 0.041 - 2.398, *p* = 0.265) regarding LRC, PFS and OS, respectively.

### Higher pre-therapeutic PD-1 expression on immune cells correlates with delayed hypoxia resolution during chemoradiation

**Figure 3:**
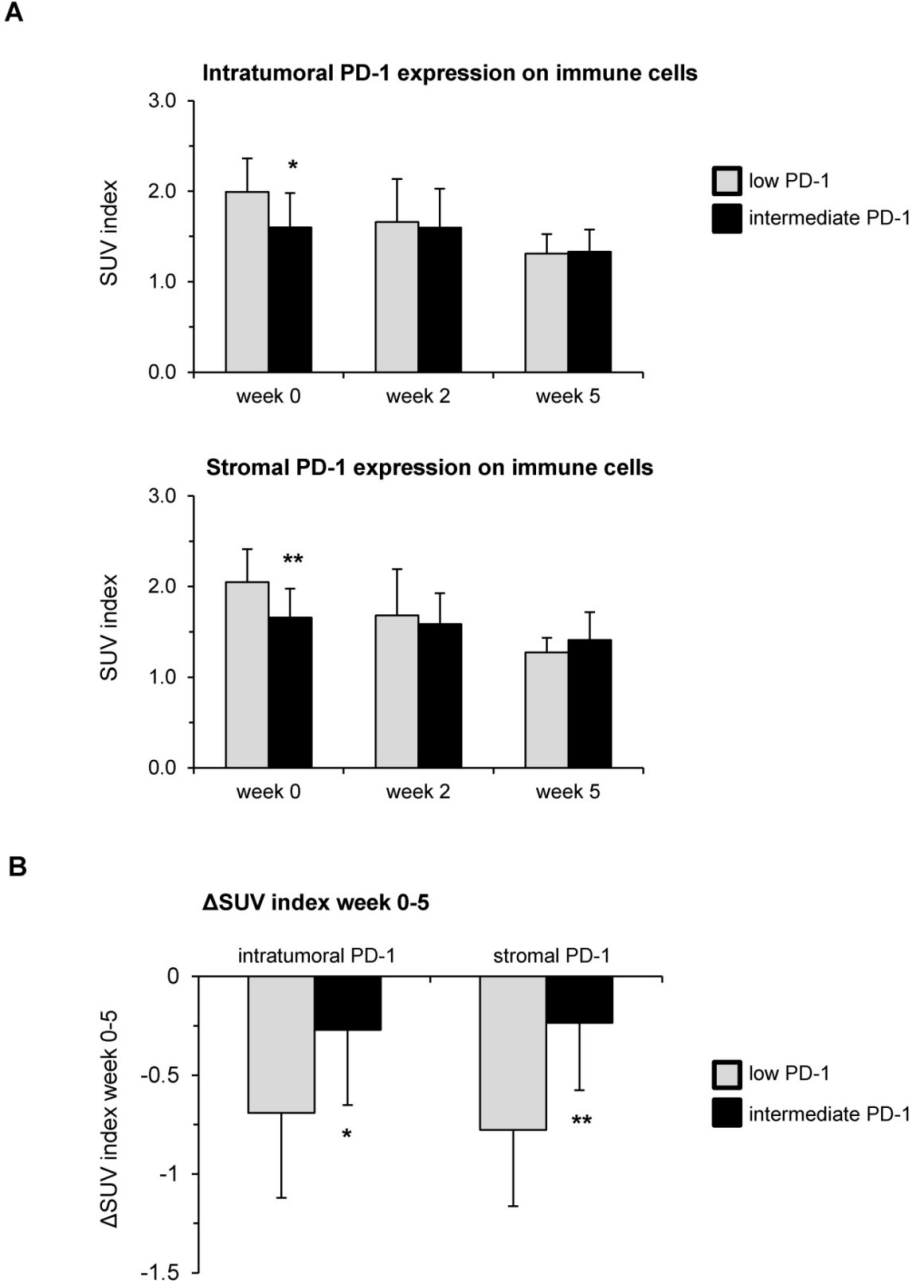
High PD-1 expression in pre-therapeutic tumor biopsies leads to reduced hypoxia resolution during chemoradiation. (**A**) SUV index values in weeks 0, 2 and 5 during chemoradiation in dependence of the pre-therapeutic PD-1 expression on immune cells. (**B**) Hypoxia resolution between weeks 0 and 5 of chemoradiation quantified by ΔSUV index values stratified by PD-1 expression. Columns represent mean values, while error bars show standard deviation. *p<0.05, **p<0.01, unpaired two-sided t-tests.

Both immune cells' PD-L1 expression as well as tumoral PD-L1 expression (TPS) were correlated with the dynamic [^18^F]FMISO PET/CT data during the course of chemoradiation using Spearman's correlation analyses. The tumor-to-muscle ratio, meaning the ratio between the maximum SUV value inside the tumor and the mean SUV in the contralateral sternocleidomastoid muscle, was used for the following analyses. Baseline hypoxia levels were significantly lower in patients with increased PD-1 expression both inside the tumor (*p* = 0.023) and within the tumor stroma (*p* = 0.004) (**Figure 3A**). However, higher levels of PD-1 were related to a decreased resolution of hypoxia between weeks 0 and 5 (**Figure 3B**). While the SUV index levels decreased by 0.27 in HNSCC patients with intermediate PD-1 expression between weeks 0 and 5 of chemoradiation, this decrease was more than twice as much (ΔSUV index = -0.69) in patients with low PD-1 expression (*p* = 0.04). The effect was even more pronounced for the stromal PD-1 expression: Hypoxia resolution between weeks 0 and 5 in patients belonging to the subgroup with intermediate pre-therapeutic PD-1 levels was significantly reduced compared to patients with low pre-therapeutic expression (ΔSUV index = -0.23 vs. -0.78, *p* = 0.001).

Notably, we neither found any correlations between the PD-L1 expression (both on immune cells and on tumor cells [TPS]) and tumor hypoxia parameters nor between the PD-L1 expression and the tested hypoxia tissue markers CAIX and HIF1α (**Table 1, Table 2**). Furthermore, the expression levels of CD34 as a marker for the microvascular density did not correspond to the PD-L1 expression. HPV-positive HNSCCs did not differ regarding their immune cells' PD-1 or PD-L1 expression compared to HPV-negative tumors (**Table 3, Table S3**).

### Absence of hypoxia resolution combined with PD-L1 expression on tumor cells (TPS ≥1%) correlates with impaired outcomes

The negative impact of absent hypoxia response between weeks 0 and 2 of chemoradiation has been described before and was observed in our study, too 6, 16, 17. Patients with early tumor hypoxia resolution defined as a negative ΔSUV index between weeks 0 and 2 of chemoradiation exhibited a significantly superior LRC (HR = 0.321, 95% CI 0.134-0.770, *p* = 0.015) and PFS (HR = 0.402, 95% CI 0.173 - 0.936, *p* = 0.043) compared to patients with increased tumor hypoxia between weeks 0 and 2 (**Figure S2**).

**Figure 4:**
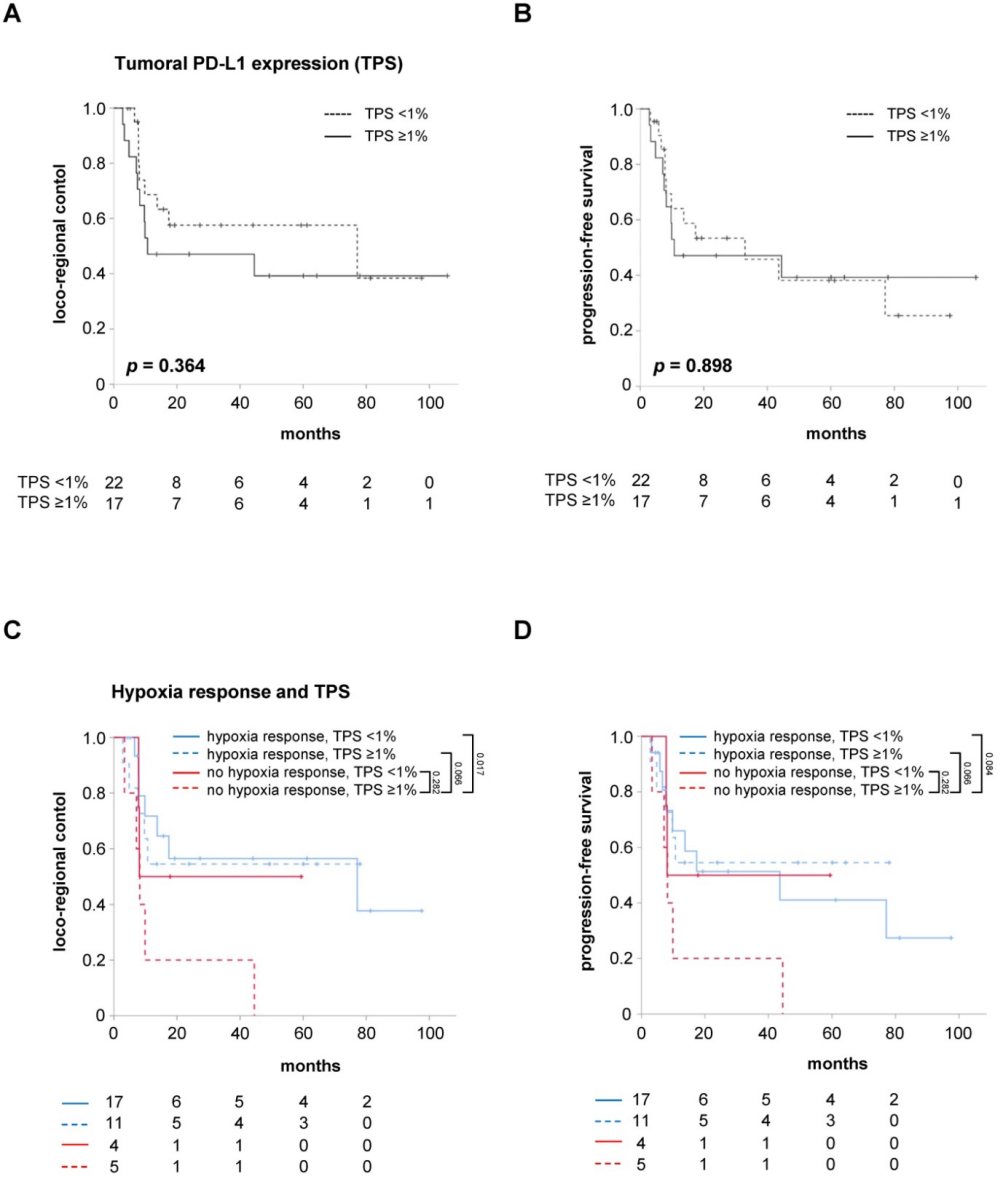
Absent early hypoxia response and high tumoral PD-L1 expression go along with worse LRC and PFS. LRC (**A**) and PFS (**B**) in HNSCC patients after chemoradiation according to the TPS as marker for the tumor cells' PD-L1 expression. Kaplan-Meier curves for LRC (**C**) and PFS (**D**) separated by early hypoxia resolution (between weeks 0 and 2) and TPS. P values are derived from log-rank tests.

However, the relevance of the TPS as a marker for the tumor cells' PD-L1 expression regarding the response prediction in hypoxia-resolving and non-resolving HNSCCs is unknown. While the pre-therapeutic tumoral PD-L1 expression alone, using a cut-off value of TPS <1%, did not influence LRC (HR = 1.515, 95% CI 0.614 - 3.738, *p* = 0.367) and PFS (HR = 1.056, 95% CI 0.455 - 2.450, *p* = 0.899) in the study cohort, patients with both a lack of early hypoxia response between weeks 0 and 2 and high TPS were found to have significantly reduced LRC (HR = 3.374, 95% CI 1.192 - 9.548, *p* = 0.022) and a trend towards reduced PFS (HR = 2.752, 95% CI 0.992 - 7.631, *p* = 0.052) (**Figure 4, Figure S3**). The median PFS of these patients amounted to only 8.2 months, which was considerably lower than the median PFS of 43.6 months for the remaining patients exhibiting either early hypoxia resolution or a TPS less than 1%.

Interestingly, a small group of patients with persistent tumor hypoxia but negative TPS (TPS <1%) exhibited statistically comparable LRC (HR = 1.214, 95% CI 0.268 - 5.496, *p* = 0.801) and PFS (HR = 0.989, 95% CI 0.222 - 4.398, *p* = 0.988) to HNSCC patients with early hypoxia resolution during chemoradiation. In contrast, positive tumoral PD-L1 expression (TPS ≥1%) was not found to compromise LRC (HR = 1.186, 95% CI 0.375 - 3.755, *p* = 0.772) or PFS (HR = 0.846, 95% CI 0.282 - 2.538, *p* = 0.766) of patients with early resolution of tumor-associated hypoxia during chemoradiation.

Uni- and multivariate Cox regression analyses were carried out to analyze the impact of key clinical parameters such as T and N stages as well as the HPV status on LRC and PFS (**Table 3**). Here, neither the T (HR = 1.286, 95% CI 0.683 - 2.421, *p* = 0.436) nor the N stage (HR = 0.704, 95% CI 0.370 - 1.338, *p* = 0.284) influenced LRC in our cohort. As demonstrated previously, HNSCC patients with positive HPV status had improved LRC (HR = 7.526, 95% CI 1.012 - 55.966, *p* = 0.049) and PFS (HR = 4.318, 95% CI 1.017 - 18.329, *p* = 0.047) compared to HPV-negative patients 31. While the tumor-to-muscle values in weeks 0, 2 and 5 were not associated with LRC or PFS, early tumor hypoxia response, defined as negative ΔSUV index between week 0 and 2, was found to significantly deteriorate LRC (HR = 3.111, 95% CI 1.299 - 7.452, *p* = 0.011) and PFS (HR = 2.487, 95% CI 1.068 - 5.749, *p* = 0.035). While the tumoral PD-L1 expression (*i.e.* TPS) alone did not influence LRC or PFS, patients with absent early hypoxia response and positive TPS exhibited significantly reduced LRC (HR = 3.374, 95% CI 1.192 - 9.548, *p* = 0.022) and showed a trend towards impaired PFS (HR = 2.752, 95% CI 0.992 - 7.631, *p* = 0.052). In the multivariate analysis, only the combination of absent early hypoxia response and positive TPS remained a significant prognosticator for LRC (HR = 3.374, 95% CI 1.192 - 9.548, *p* = 0.022) and, with borderline significance, for PFS (HR = 2.752, 95% CI 0.992 - 7.631, *p* = 0.052).

## Discussion

In this prospective imaging trial, we confirmed the results of other groups regarding the negative impact of absent hypoxia resolution between week 0 and 2 of chemoradiation. Interestingly, we showed that patients with persistent tumor-associated hypoxia in week 2 of chemoradiation and high pre-therapeutic PD-L1 expression on tumor cells (quantified by a TPS ≥1%) exhibited reduced LRC and PFS. On the other side, increased tumoral PD-L1 expression did not impair the outcomes of patients with early hypoxia resolution.

The observation that the subgroup of HNSCC patients with an absent early hypoxia response and tumoral PD-L1 expression exhibited the worst prognosis regarding LRC and PFS is in line with a recently proposed hypoxia-transcriptional classifier that identified 3 patient subgroups with different hypoxia-immune phenotypes: A hypoxia_low_/immune_high_, a hypoxia_high_/immune_low_ and a mixed phenotype 35. In that study, it could be shown that each group had different biological characteristics with the worst outcome for the hypoxia_high_/immune_low_ subgroup. Our data regarding the detrimental prognosis of patients with no hypoxia response and high TPS values backs up the concept of a hypoxia-immune marker-based prognostic score in a prospective clinical trial.

There are conflicting data regarding the prognostic value of tumoral PD-L1 expression in HNSCC patients 36-40. Balermpas et al. observed a positive impact of high PD-L1 expression on the survival in a large, multicenter cohort of patients with locally advanced HNSCC undergoing adjuvant chemoradiation 37. Interestingly, Kim and colleagues reported that only high PD-L1 expression on immune cells but not on tumor cells improved survival rates in HNSCC patients who underwent surgical resection 38. Another study described PD-L1 overexpression on tumor cells as a negative prognosticator for HNSCC patients treated by adjuvant chemoradiation 41. The conflicting results may be related to the heterogeneous study populations in some studies in which definitive and adjuvant radiation treatment as well as chemoradiation and radiotherapy alone were mixed. Another reason for the varying findings may be due to the usage of differing cut-off values for PD-L1 expression as well as differing antibodies and staining protocols used in these studies. As PD-L1 is known to be heterogeneously expressed within tumors, cohort analyses of patients with definitive chemoradiation are more complicated due to the small tumor samples that are routine available from biopsies of these patients 42. However, examinations in patients undergoing definitive chemoradiation are required to assess potential interactions between the hypoxic microenvironment as detected by [^18^F]FMISO imaging and PD-1/PD-L1 expression clinically.

The negative prognostic role of the TPS in our cohort must be distinguished from the predictive role of the TPS regarding checkpoint inhibitor treatment. In the Keynote-040-study, patients with a TPS ≥50% significantly improved from pembrolizumab, whereas patients with a TPS <50% did not 29. The Checkmate 141-trial demonstrated that patients exhibiting a tumor cell PD-L1 expression >1% benefited to a greater extent from nivolumab treatment than patients with PD-L1 negative tumor cells 26, 43. The significantly impaired outcomes of HNSCC patients with missing early hypoxia resolution and positive TPS in combination with the reported predictive role of PD-L1 regarding checkpoint inhibitor therapy gives a rationale to investigate checkpoint inhibitor treatment as a supplement to local radiation therapy for this subgroup. So far, treatment escalation strategies for patients with pronounced tumor hypoxia or lack of early hypoxia resolution during chemoradiation are mostly based on the escalation of the radiation dose to hypoxic subvolumes using intensity-modulated radiotherapy or concomitant application of hypoxia modifiers such as nimorazole, hyperbaric oxygen or hyperthermia 44-50.

A lack of hypoxia response within the first 2 weeks of chemoradiation suggests a continued immunosuppressive microenvironment, which may be at least partly resolved by immune checkpoint inhibitor administration alone or in combination with hypoxic modifiers such as nimorazole. The fact that patients with no hypoxia resolution but low tumoral PD-L1 expression (TPS <1%) had comparable outcomes to patients with hypoxia resolution suggests that missing early hypoxia resolution alone may be an inferior predictor for treatment outcome compared to the combination of hypoxia dynamics and tumoral PD-L1 status. However, the comparable outcome of these patients should be interpreted with caution due to the relatively small sample size in this subgroup, showing the importance of further prospective imaging trials with combined immunohistochemistry.

*Vice versa*, tumoral PD-L1 expression had no impact on the favorable outcome of patients whose tumor-associated hypoxia decreases between week 0 and week 2 of chemoradiation. As hypoxia upregulates PD-L1, declining tumor hypoxia during treatment could lead to reduced tumoral PD-L1 expression and could abrogate the PD-1/PD-L1 immune suppression pathway, leading to improved anti-tumor activity of tumor-infiltrating lymphocytes 51. Treatment-induced resolution of tumor-associated hypoxia could therefore restore the anti-tumor activity of already present lymphocytes.

While other studies have shown correlations between HIF1 and PD-L1 expression in some tumor entities, we did not find associations between the tissue hypoxia biomarkers and the PD-1/PD-L1 expression, which may be related to the small tumor biopsies used in our analysis 52, 53. Tissue hypoxia is known to be heterogeneously distributed within the tumor, and tumor biopsies may therefore not fully represent the hypoxic subvolumes. Therefore, hypoxia imaging that provides spatial hypoxia information throughout the entire tumor could have advantages over histological hypoxia analyses of small tissue samples in the definitive treatment setting 54. Additionally, we observed that the tumoral PD-L1 expression was independent of the HPV status in our cohort, which is in line with a previous study of Wang and colleagues 55.

The observed correlation of the intratumoral PD-1 expression with impaired hypoxia resolution during chemoradiation warrants mechanistical analyses to further analyze this interaction. There are only few data regarding the interplay between tumor hypoxia and the PD-1/PD-L1 axis 51. In an animal study, PD-1 expression was found unaffected by hypoxia 51. As PD-1 is a T cell exhaustion marker, the reduced hypoxia resolution may be related to impaired anti-tumor activity of the infiltrating T cells and therefore decreased tumor downsizing 56. On the other hand, the diminished hypoxia resolution in patients with higher PD-1 expression could be a resolute of the lower baseline tumor-associated hypoxia levels in this subgroup. Sampling of tumor biopsies immediately after completion of chemoradiation would be highly valuable in order to investigate this relationship more deeply; however, this was not feasible due to ethical concerns in our trial.

Although studies of the PD-1/PD-L1 expression by immunohistochemistry are usually limited to the pre-treatment biopsies, repeat analyses during the course of treatment may add valuable information. In this regard, non-invasive PD-1/PD-L1 monitoring by immuno-PET using radiolabeled PD-1- and PD-L1-antibodies may allow investigation of the observed correlation between PD-1/PD-L1 expression and impaired hypoxia resolution directly and longitudinally during chemoradiation 57, 58. For instance, Kikuchi and colleagues could show in an animal model that non-invasive monitoring of PD-L1 expression during HNSCC radiotherapy was feasible using a Zr-89 labeled anti-mouse PD-L1 antibody 59. Notably, functional imaging of PD-1 is considerably less often performed than imaging of PD-L1 58; however, successful imaging of PD-1 has also been achieved *in vivo* 57, 60. The potential advantages of functional immuno-PET imaging compared to tumor biopsies include the non-invasive character, the possibility for real-time measurements and the coverage of the whole tumor including distant metastases, thereby addressing the well-known heterogeneity of metastatic malignancies.

Within the different [^18^F]FMISO PET imaging trials, different quantification techniques for tumor hypoxia parameters were used. We considered subvolumes inside the tumor as hypoxic, if the ratio of tumor [^18^F]FMISO SUV to mean SUV in the contralateral sternocleidomastoid muscle was above 1.4. While some groups applied the same ratio 3; other groups have used different cut-off values such as 1.6 16. Additionally, we have used the contralateral sternocleidomastoid muscle as background reference, while others used the contralateral carotid artery, the left ventricle or the cerebellum. In our analysis, the maximum SUV value inside the tumor was measured irrespectively of the voxel numbers in which this SUV value was present. This was different in another [^18^F]FMISO trial, where the mean SUV value within the 5 × 5 × 5 voxels of highest activity was considered as SUV_peak_ value.

Despite the fact that our exploratory analysis is based on a prospective trial, there are some limitations to our analysis. We did not perform internal validations due to the relatively small sample size, which is why a validation in an external independent cohort is warranted to confirm our findings. We did not adjust for multiple testing, as our analysis was an exploratory analysis aiming to generate hypotheses regarding the interaction between tumor hypoxia dynamics and the PD-1/PD-L1 axis. As our study included only patients receiving definitive chemoradiation, only small tumor biopsies were available for pathological analyzes which may not be fully representative of the complex and heterogeneous tumor microenvironment, and no longitudinal analyses of PD-1/PD-L1 expression were possible due to the lack of tissue during the course of treatment. Additionally, the limited tumor material did not allow for further histopathological analyses regarding other parameters for the tumor immune microenvironment such as regulatory T cells, myeloid-derived suppressor cells and tumor-associated macrophages.

## Conclusion

Patients with persistent tumor-associated hypoxia and high PD-L1 expression (TPS ≥1%) on tumor cells exhibited considerably inferior outcomes, while the favorable outcome of patients with early hypoxia response was independent of the tumoral PD-L1 expression. In contrast, low tumoral PD-L1 expression (TPS <1%) did not further improve the superior outcome of patients with tumor hypoxia resolution. Although our results should be interpreted with caution due to the incompletely understood underlying mechanisms and the missing validation in an independent cohort, our findings form a foundation to investigate the combination of hypoxic modification and immune checkpoint inhibitors as well as radiation dose escalation to hypoxic sub-volumes for the unfavorable subgroup, helping to bring personalized radiotherapy treatment into clinical routine.

## Supplementary Material

Supplementary figures and tables.

## Figures and Tables

**Table 1:** Spearman's correlations between immune biomarkers and tumor hypoxia in week 0, 2 and 5 during chemoradiation. Spearman's ρ and p values are indicated in the table

		Tumor volume	Hypoxic subvolume	SUV index w0	SUV index w2	SUV index w5	ΔSUV index w0-w2	ΔSUV index w0-w5
PD-L1 tumor	ρ	0.142	0.283	-0.009	-0.019	0.188	-0.093	0.065
	p	0.395	0.081	0.957	0.910	0.271	0.584	0.707
PD-L1 stroma	ρ	-0.074	-0.023	-0.075	0.011	0.108	0.194	0.157
	p	0.661	0.890	0.649	0.949	0.532	0.250	0.361
TPS	ρ	-0.084	-0.100	-0.127	0.207	0.079	0.324	0.108
	p	0.618	0.545	0.442	0.218	0.646	0.050	0.531

Abbreviations: PD-L1 = Programmed death-ligand 1, TPS = Tumor proportional score, SUV = standardized uptake value, wk = week.

**Table 2:** Spearman's correlations between immune and hypoxia biomarkers. Spearman's ρ and p values are indicated in the table

		HIF1α	CAIX	CD34
PD-L1 tumor	ρ	0.240	0.181	0.000
	p	0.141	0.271	1.000
PD-L1 stroma	ρ	-0.025	0.158	0.127
	p	0.880	0.337	0.441
TPS	ρ	0.100	0.297	-0.062
	p	0.546	0.067	0.706

Abbreviations: PD-L1 = Programmed death-ligand 1, TPS = Tumor proportional score, HIF1α = Hypoxia-inducible factor-1α, CAIX = Carbonic anhydrase IX.

**Table 3:** Cox regression analysis of clinical and pathologic parameters in terms of LRC and PFS

	LRC		PFS	
**Univariate**	**HR**	***p*-value**	**HR**	***p*-value**
T stage (continuous)	1.286	0.436	1.089	0.760
N stage (continuous)	0.704	0.284	0.804	0.504
HPV-negative	7.526	0.049	4.318	0.047
TPS ≥1%	1.515	0.367	1.056	0.899
TPS ≥10%	1.112	0.804	0.930	0.861
TPS ≥50%	1.042	0.956	0.862	0.843
SUV index wk0 (tumor-to-muscle) ≥1.89 (median)	1.243	0.612	1.520	0.312
SUV index wk2 (tumor-to muscle) ≥1.51 (median)	1.883	0.158	1.702	0.213
SUV index wk5 (tumor-to muscle) ≥1.31 (median)	0.715	0.460	0.450	0.725
No early hypoxia response (ΔSUV index wk0-2 ≥0)	3.111	0.011	2.487	0.035
No early hypoxia response + TPS ≥1%	3.374	0.022	2.752	0.052
**Multivariate (backward elimination with likelihood ratio tests)**				
HPV-negative	2.936	0.305	1.637	0.517
No early hypoxia resolution (ΔSUV index wk0-2 ≥0)	1.217	0.799	0.996	0.996
No early hypoxia resolution + TPS ≥1%	3.374	0.022	2.752	0.052

Abbreviations: HPV = Human papillomavirus, TPS = Tumor proportional score, wk = week, SUV = standardized uptake value.

## References

[1] Bray F, Ferlay J, Soerjomataram I, Siegel RL, Torre LA, Jemal A (2018). Global cancer statistics 2018: GLOBOCAN estimates of incidence and mortality worldwide for 36 cancers in 185 countries. CA Cancer J Clin.

[2] Pulte D, Brenner H (2010). Changes in survival in head and neck cancers in the late 20th and early 21st century: a period analysis. Oncologist.

[3] Mortensen LS, Johansen J, Kallehauge J, Primdahl H, Busk M, Lassen P (2012). FAZA PET/CT hypoxia imaging in patients with squamous cell carcinoma of the head and neck treated with radiotherapy: results from the DAHANCA 24 trial. Radiother Oncol.

[4] Vaupel P, Thews O, Hoeckel M (2001). Treatment resistance of solid tumors: role of hypoxia and anemia. Med Oncol.

[5] Gray LH, Conger AD, Ebert M, Hornsey S, Scott OC (1953). The concentration of oxygen dissolved in tissues at the time of irradiation as a factor in radiotherapy. Br J Radiol.

[6] Zips D, Zophel K, Abolmaali N, Perrin R, Abramyuk A, Haase R (2012). Exploratory prospective trial of hypoxia-specific PET imaging during radiochemotherapy in patients with locally advanced head-and-neck cancer. Radiother Oncol.

[7] Stadler P, Feldmann HJ, Creighton C, Kau R, Molls M (1998). Changes in tumor oxygenation during combined treatment with split-course radiotherapy and chemotherapy in patients with head and neck cancer. Radiother Oncol.

[8] Koukourakis MI, Giatromanolaki A, Sivridis E, Simopoulos C, Turley H, Talks K (2002). Hypoxia-inducible factor (HIF1A and HIF2A), angiogenesis, and chemoradiotherapy outcome of squamous cell head-and-neck cancer. Int J Radiat Oncol Biol Phys.

[9] Grosu AL, Souvatzoglou M, Röper B, Dobritz M, Wiedenmann N, Jacob V (2007). Hypoxia imaging with FAZA-PET and theoretical considerations with regard to dose painting for individualization of radiotherapy in patients with head and neck cancer. Int J Radiat Oncol Biol Phys.

[10] Vaupel P, Höckel M, Mayer A (2007). Detection and characterization of tumor hypoxia using pO2 histography. Antioxid Redox Signal.

[11] Wiedenmann N, Grosu AL, Büchert M, Rischke HC, Ruf J, Bielak L (2020). The utility of multiparametric MRI to characterize hypoxic tumor subvolumes in comparison to FMISO PET/CT. Consequences for diagnosis and chemoradiation treatment planning in head and neck cancer. Radiother Oncol.

[12] Rajendran JG, Schwartz DL, O'Sullivan J, Peterson LM, Ng P, Scharnhorst J (2006). Tumor hypoxia imaging with [F-18] fluoromisonidazole positron emission tomography in head and neck cancer. Clin Cancer Res.

[13] Rasey JS, Koh WJ, Evans ML, Peterson LM, Lewellen TK, Graham MM (1996). Quantifying regional hypoxia in human tumors with positron emission tomography of [18F]fluoromisonidazole: a pretherapy study of 37 patients. Int J Radiat Oncol Biol Phys.

[14] Kadrmas DJ, Hoffman JM (2013). Methodology for quantitative rapid multi-tracer PET tumor characterizations. Theranostics.

[15] Jacobson O, Chen X (2013). Interrogating tumor metabolism and tumor microenvironments using molecular positron emission tomography imaging. Theranostic approaches to improve therapeutics. Pharmacol Rev.

[16] Lock S, Perrin R, Seidlitz A, Bandurska-Luque A, Zschaeck S, Zophel K (2017). Residual tumour hypoxia in head-and-neck cancer patients undergoing primary radiochemotherapy, final results of a prospective trial on repeat FMISO-PET imaging. Radiother Oncol.

[17] Wiedenmann NE, Bucher S, Hentschel M, Mix M, Vach W, Bittner MI (2015). Serial [18F]-fluoromisonidazole PET during radiochemotherapy for locally advanced head and neck cancer and its correlation with outcome. Radiother Oncol.

[18] Daşu A, Toma-Daşu I, Karlsson M (2005). The effects of hypoxia on the theoretical modelling of tumour control probability. Acta Oncol.

[19] Bittner MI, Grosu AL (2013). Hypoxia in head and neck tumors: Characteristics and development during therapy. Front Oncol.

[20] Baumann M, Krause M, Overgaard J, Debus J, Bentzen SM, Daartz J (2016). Radiation oncology in the era of precision medicine. Nat Rev Cancer.

[21] Noman MZ, Hasmim M, Messai Y, Terry S, Kieda C, Janji B (2015). Hypoxia: A key player in antitumor immune response. A review in the theme: Cellular responses to hypoxia. Am J Physiol Cell Physiol.

[22] Eckert F, Zwirner K, Boeke S, Thorwarth D, Zips D, Huber SM (2019). Rationale for combining radiotherapy and immune checkpoint inhibition for patients with hypoxic tumors. Front Immunol.

[23] Hasmim M, Noman MZ, Messai Y, Bordereaux D, Gros G, Baud V (2013). Cutting edge: Hypoxia-induced Nanog favors the intratumoral infiltration of regulatory T cells and macrophages via direct regulation of TGF-β1. J Immunol.

[24] Jiang X, Wang J, Deng X, Xiong F, Ge J, Xiang B (2019). Role of the tumor microenvironment in PD-L1/PD-1-mediated tumor immune escape. Mol Cancer.

[25] Westendorf AM, Skibbe K, Adamczyk A, Buer J, Geffers R, Hansen W (2017). Hypoxia enhances immunosuppression by inhibiting CD4+ effector T cell function and promoting Treg activity. Cell Physiol Biochem.

[26] Ferris RL, Blumenschein G Jr, Fayette J, Guigay J, Colevas AD, Licitra L (2016). Nivolumab for recurrent squamous-cell carcinoma of the head and neck. N Engl J Med.

[27] Seiwert TY, Burtness B, Mehra R, Weiss J, Berger R, Eder JP (2016). Safety and clinical activity of pembrolizumab for treatment of recurrent or metastatic squamous cell carcinoma of the head and neck (KEYNOTE-012): an open-label, multicentre, phase 1b trial. Lancet Oncol.

[28] Burtness B, Harrington KJ, Greil R, Soulières D, Tahara M, de Castro G Jr (2019). Pembrolizumab alone or with chemotherapy versus cetuximab with chemotherapy for recurrent or metastatic squamous cell carcinoma of the head and neck (KEYNOTE-048): a randomised, open-label, phase 3 study. Lancet.

[29] Cohen EEW, Soulières D, Le Tourneau C, Dinis J, Licitra L, Ahn MJ (2019). Pembrolizumab versus methotrexate, docetaxel, or cetuximab for recurrent or metastatic head-and-neck squamous cell carcinoma (KEYNOTE-040): a randomised, open-label, phase 3 study. Lancet.

[30] von der Grün J, Rödel F, Brandts C, Fokas E, Guckenberger M, Rödel C (2019). Targeted therapies and immune-checkpoint inhibition in head and neck squamous cell carcinoma: Where do we stand today and where to go?. Cancers.

[31] Nicolay NH, Wiedenmann N, Mix M, Weber WA, Werner M, Grosu AL (2019). Correlative analyses between tissue-based hypoxia biomarkers and hypoxia PET imaging in head and neck cancer patients during radiochemotherapy-results from a prospective trial. Eur J Nucl Med Mol Imaging.

[32] Wiedenmann N, Bunea H, Rischke HC, Bunea A, Majerus L, Bielak L (2018). Effect of radiochemotherapy on T2* MRI in HNSCC and its relation to FMISO PET derived hypoxia and FDG PET. Radiat Oncol.

[33] Grosu AL, Lachner R, Wiedenmann N, Stärk S, Thamm R, Kneschaurek P (2003). Validation of a method for automatic image fusion (BrainLAB System) of CT data and 11C-methionine-PET data for stereotactic radiotherapy using a LINAC: first clinical experience. Int J Radiat Oncol Biol Phys.

[34] Bittner MI, Wiedenmann N, Bucher S, Hentschel M, Mix M, Weber WA (2013). Exploratory geographical analysis of hypoxic subvolumes using 18F-MISO-PET imaging in patients with head and neck cancer in the course of primary chemoradiotherapy. Radiother Oncol.

[35] Brooks JM, Menezes AN, Ibrahim M, Archer L, Lal N, Bagnall CJ (2019). Development and validation of a combined hypoxia and immune prognostic classifier for head and neck cancer. Clin Cancer Res.

[36] Müller T, Braun M, Dietrich D, Aktekin S, Höft S, Kristiansen G (2017). PD-L1: a novel prognostic biomarker in head and neck squamous cell carcinoma. Oncotarget.

[37] Balermpas P, Rödel F, Krause M, Linge A, Lohaus F, Baumann M (2017). The PD-1/PD-L1 axis and human papilloma virus in patients with head and neck cancer after adjuvant chemoradiotherapy: A multicentre study of the German Cancer Consortium Radiation Oncology Group (DKTK-ROG). Int J Cancer.

[38] Kim HR, Ha SJ, Hong MH, Heo SJ, Koh YW, Choi EC (2016). PD-L1 expression on immune cells, but not on tumor cells, is a favorable prognostic factor for head and neck cancer patients. Sci Rep.

[39] Lin YM, Sung WW, Hsieh MJ, Tsai SC, Lai HW, Yang SM (2015). High PD-L1 expression correlates with metastasis and poor prognosis in oral squamous cell carcinoma. PLoS One.

[40] Straub M, Drecoll E, Pfarr N, Weichert W, Langer R, Hapfelmeier A (2016). CD274/PD-L1 gene amplification and PD-L1 protein expression are common events in squamous cell carcinoma of the oral cavity. Oncotarget.

[41] Yang F, Zeng Z, Li J, Zheng Y, Wei F, Ren X (2018). PD-1/PD-L1 axis, rather than high-mobility group alarmins or CD8+ tumor-infiltrating lymphocytes, is associated with survival in head and neck squamous cell carcinoma patients who received surgical resection. Front Oncol.

[42] McLaughlin J, Han G, Schalper KA, Carvajal-Hausdorf D, Pelekanou V, Rehman J (2016). Quantitative assessment of the heterogeneity of PD-L1 expression in non-small-cell lung cancer. JAMA Oncol.

[43] Ferris RL, Blumenschein G Jr, Fayette J, Guigay J, Colevas AD, Licitra L (2018). Nivolumab vs investigator's choice in recurrent or metastatic squamous cell carcinoma of the head and neck: 2-year long-term survival update of CheckMate 141 with analyses by tumor PD-L1 expression. Oral Oncol.

[44] Lee NY, Mechalakos JG, Nehmeh S, Lin Z, Squire OD, Cai S (2008). Fluorine-18-labeled fluoromisonidazole positron emission and computed tomography-guided intensity-modulated radiotherapy for head and neck cancer: a feasibility study. Int J Radiat Oncol Biol Phys.

[45] Thorwarth D, Eschmann SM, Paulsen F, Alber M (2007). Hypoxia dose painting by numbers: a planning study. Int J Radiat Oncol Biol Phys.

[46] Welz S, Monnich D, Pfannenberg C, Nikolaou K, Reimold M, La Fougere C (2017). Prognostic value of dynamic hypoxia PET in head and neck cancer: Results from a planned interim analysis of a randomized phase II hypoxia-image guided dose escalation trial. Radiother Oncol.

[47] Kaanders JH, Bussink J, van der Kogel AJ (2002). ARCON: a novel biology-based approach in radiotherapy. Lancet Oncol.

[48] Overgaard J, Eriksen JG, Nordsmark M, Alsner J, Horsman MR (2005). Plasma osteopontin, hypoxia, and response to the hypoxia sensitiser nimorazole in radiotherapy of head and neck cancer: results from the DAHANCA 5 randomised double-blind placebo-controlled trial. Lancet Oncol.

[49] Janssens GO, Rademakers SE, Terhaard CH, Doornaert PA, Bijl HP, van den Ende P (2012). Accelerated radiotherapy with carbogen and nicotinamide for laryngeal cancer: results of a phase III randomized trial. J Clin Oncol.

[50] Horsman MR, Overgaard J (2007). Hyperthermia: a potent enhancer of radiotherapy. Clin Oncol (R Coll Radiol).

[51] Noman MZ, Desantis G, Janji B, Hasmim M, Karray S, Dessen P (2014). PD-L1 is a novel direct target of HIF-1α, and its blockade under hypoxia enhanced MDSC-mediated T cell activation. J Exp Med.

[52] Dai X, Pi G, Yang S-L, Chen GG, Liu L-P, Dong H-H (2018). Association of PD-L1 and HIF-1α coexpression with poor prognosis in hepatocellular carcinoma. Transl Oncol.

[53] Chang YL, Yang CY, Lin MW, Wu CT, Yang PC (2016). High co-expression of PD-L1 and HIF-1α correlates with tumour necrosis in pulmonary pleomorphic carcinoma. Eur J Cancer.

[54] Serganova I, Doubrovin M, Vider J, Ponomarev V, Soghomonyan S, Beresten T (2004). Molecular imaging of temporal dynamics and spatial heterogeneity of hypoxia-inducible factor-1 signal transduction activity in tumors in living mice. Cancer Res.

[55] Wang J, Sun H, Zeng Q, Guo XJ, Wang H, Liu HH (2019). HPV-positive status associated with inflamed immune microenvironment and improved response to anti-PD-1 therapy in head and neck squamous cell carcinoma. Sci Rep.

[56] Wherry EJ, Kurachi M (2015). Molecular and cellular insights into T cell exhaustion. Nat Rev Immunol.

[57] Hettich M, Braun F, Bartholoma MD, Schirmbeck R, Niedermann G (2016). High-resolution PET imaging with therapeutic antibody-based PD-1/PD-L1 checkpoint tracers. Theranostics.

[58] Lütje S, Feldmann G, Essler M, Brossart P, Bundschuh RA (2020). Immune checkpoint imaging in oncology - a game changer towards personalized immunotherapy?. J Nucl Med.

[59] Kikuchi M, Clump DA, Srivastava RM, Sun L, Zeng D, Diaz-Perez JA (2017). Preclinical immunoPET/CT imaging using Zr-89-labeled anti-PD-L1 monoclonal antibody for assessing radiation-induced PD-L1 upregulation in head and neck cancer and melanoma. Oncoimmunology.

[60] Natarajan A, Mayer AT, Reeves RE, Nagamine CM, Gambhir SS (2017). Development of novel immunoPET tracers to image human PD-1 checkpoint expression on tumor-infiltrating lymphocytes in a humanized mouse model. Mol Imaging Biol.

