# Supplementary material for: Hypoxia dynamics on FMISO-PET in combination with PD-1/PD-L1 expression has an impact on the clinical outcome of patients with Head-and-neck Squamous Cell Carcinoma undergoing Chemoradiation
Source: Theranostics. 2020 Jul 23;10(20):9395–406. doi: 10.7150/thno.48392 (PMC7415814; doi:10.7150/thno.48392)
Supplement: Supplementary file 1 — Supplementary figures and tables. [file thnov10p9395s1.pdf]

Supplementary table 1: Patient characteristics including demographic and tumor-related parameters (n=49).

Patient characteristics		
Age (years)		
Median	60	
Minimum	34	
Maximum	78	
Gender	**n**	**%**
Male	44	89.8
Female	5	10.2
Tumor location		
Oral cavity	2	4.1
Oropharynx	19	38.8
Hypopharynx	12	24.5
Larynx	7	14.3
Multi-level	9	18.4
Tumor extent		
T1	1	2.0
T2	4	8.2
T3	14	28.6
T4	30	61.2
Nodal status		
N0	4	8.2
N1	1	2.0
N2a	0	0.0
N2b	9	18.4
N2c	35	71.4
HPV status		
HPV positive	9	18.4
HPV negative	40	81.6

Abbreviations: HPV=human papillomavirus

Supplementary table 2: Description of antibodies used for immunohistochemical stains and corresponding antigen retrieval.

Antigen	Clone	Company	Dilution	Antigen retrieval
PD-1	NAT105	Roche/Ventana	Prediluted	Tris-EDTA buffer pH 9
PD-L1	SP263	Roche/Ventana	Prediluted	Tris-EDTA buffer pH 9
CAIX	C48E	Cell Signaling	1:300	pH 6.1 citrate buffer
CD34	QBEnd10 (IR632)	DAKO	Prediluted	pH 6.1 citrate buffer
HIF1α	MAB1935	R&D	1:100	pH 6.1 citrate buffer

Supplementary table 3: Tests for potential differences regarding the PD-1 and PD-L1 expression (including the tumoral PD-L1 expression [TPS]) depending on the HPV status. *P* values of the appropriate tests are indicated.

		PD-1 tumor	PD-1 stromal	PD-L1 tumor	PD-L1 stromal	TPS
HPV status	p	0.805[1]	0.589[1]	0.718[2]	0.582[2]	0.635[2]

[1] Chi-square test, [2] Mann-Whitney-U test

Supplementary figure 1. OS in HNSCC patients undergoing chemoradiation is independent from pre-therapeutic PD-L1 and PD-1 expression on intratumoral immune cells. OS of HNSCC patients receiving chemoradiation stratified by PD-L1 (**A**) and PD-1 (**B**) expression. *P* values are derived from log-rank tests.

A

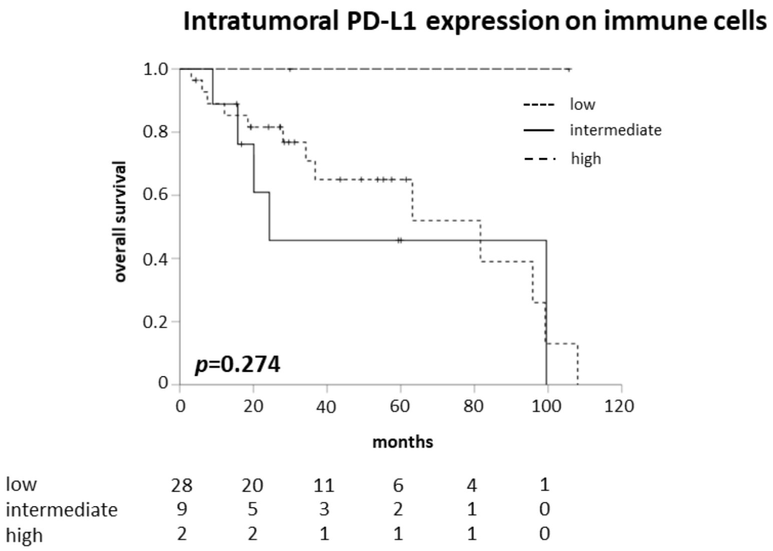

B

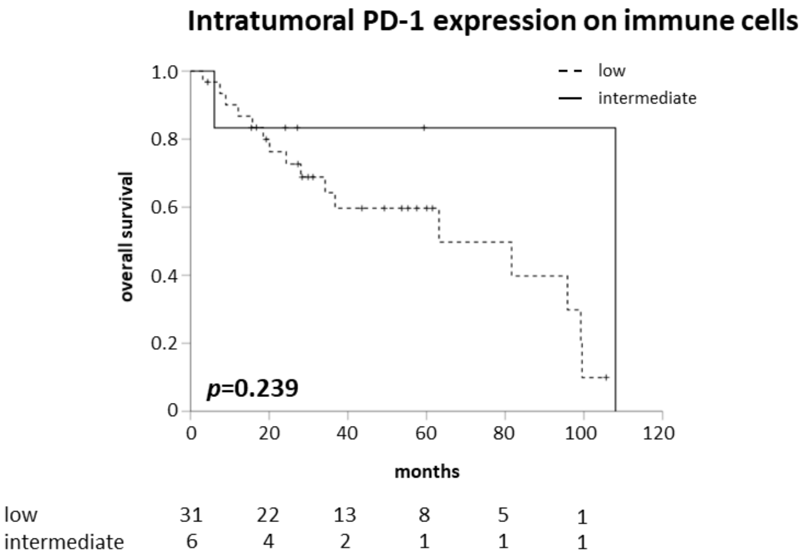

Supplementary figure 2. Early hypoxia response leads to improved LRC and PFS in HNSCC patients receiving definitive chemoradiation. LRC (**A**) and PFS (**B**) of HNSCC patients stratified by tumor hypoxia resolution between week 0 and 2 of chemoradiation. *P* values are derived from log-rank tests.

A

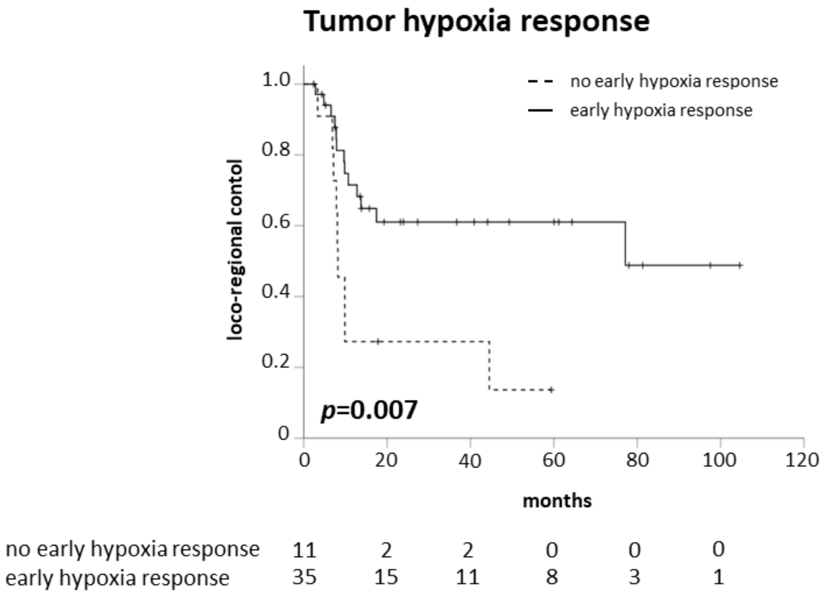

B

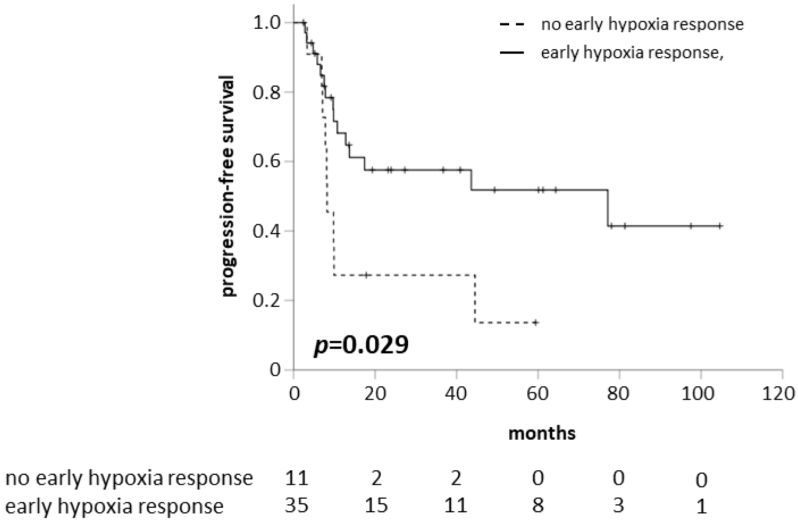

Supplementary figure 3. Persistent tumor-associated hypoxia and high TPS result in worse LRC and PFS. LRC (**A**) and PFS (**B**) of HNSCC patients with missing early hypoxia resolution (between weeks 0 and 2) and high TPS compared to the remaining patients with either present early hypoxia resolution or low TPS. *P* values are derived from log-rank tests.

A

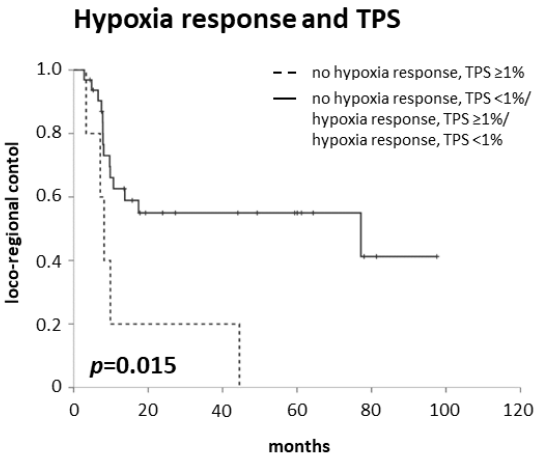

no hypoxia response, TPS ≥1%	5	1	1	0	0	0
no hypoxia response, TPS <1%/ hypoxia response, TPS ≥1%/ hypoxia response, TPS <1%	32	12	10	7	2	0

B

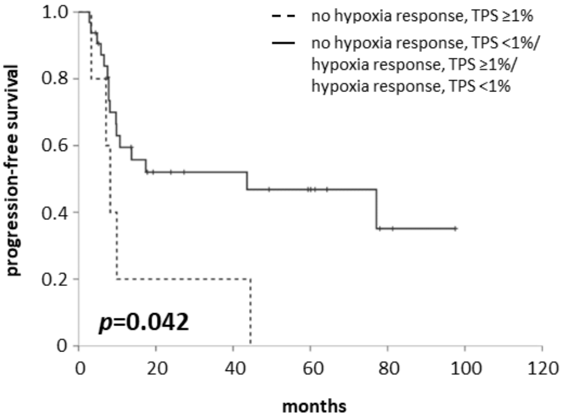

no hypoxia response, TPS ≥1%	5	1	1	0	0	0
no hypoxia response, TPS <1%/ hypoxia response, TPS ≥1%/ hypoxia response, TPS <1%	32	12	10	7	2	0
